# Optimized spray-dried conditions’ impact on fatty acid profiles and estimation of in vitro digestion of spray-dried chia/fish oil microcapsules

**DOI:** 10.1038/s41598-024-65214-x

**Published:** 2024-06-26

**Authors:** Muhammad Abdul Rahim, Joe M. Regenstein, Fahad Al-Asmari, Muhammad Imran, Mohamed Fawzy Ramadan, João Miguel F. Rocha, Imtiaz Hussain, Eliasse Zongo

**Affiliations:** 1 Department of Food Science and Nutrition, Faculty of Medicine and Allied Health Sciences, Times Institute, Multan, Pakistan; 2 Department of Food Science, Cornell University, Ithaca, NY USA; 3 Department of Food and Nutrition Sciences, College of Agricultural and Food Sciences, King Faisal University, 31982 Hofuf, Al-Ahsa Kingdom of Saudi Arabia; 4 Department of Food Science, Faculty of Life Sciences, Government College University, Faisalabad, 38000 Pakistan; 5 Department of Clinical Nutrition, Faculty of Applied Medical Sciences, Umm Al-Qura University, Makkah, Kingdom of Saudi Arabia; 6 CBQF—Centro de Biotecnologia e Química Fina—Laboratório Associado, Escola Superior de Biotecnologia, Universidade Católica Portuguesa, Rua Diogo Botelho 1327, 4169-005 Porto, Portugal; 7 LEPABE – Laboratory for Process Engineering, Environment, Biotechnology and Energy, Faculty of Engineering, University of Porto (FEUP), Rua Dr. Roberto Frias, S/N, 4200-465 Porto, Portugal; 8 ALiCE – Associate Laboratory in Chemical Engineering, Faculty of Engineering, University of Porto (FEUP), Rua Dr. Roberto Frias, s/n, 4200-465 Porto, Portugal; 9 Department of Food Science and Technology, Faculty of Agriculture, University of Poonch, Rawalakot, Azad Kashmir Pakistan; 10 Laboratoire de Recherche et d’Enseignement en Santé et Biotechnologies Animales, Université Nazi BONI, 01 BP 1091, Bobo Dioulasso, Burkina Faso

**Keywords:** Long–chain polyunsaturated fatty acids, Spray–drying, Controlled release, Chia seeds, Fish oil, Biochemistry, Biotechnology

## Abstract

Long–chain polyunsaturated fatty acids (LCPUFA) are of interest due to their potential health properties and have a significant role in reducing the risk of various chronic diseases in humans. It is commonly used as a supplement. However, lipid oxidation is an important negative factor caused by environmental, processing, and limited water solubility of LCPUFA, making them difficult to incorporate into food products. The objective of this research work was to prevent oxidation, extend shelf life, enhance the stability of fatty acids, and to achieve controlled release by preparing spray-dried powder (SDM). For spray-drying, aqueous emulsion blends were formulated using a 1:1 ratio of chia seed oil (CSO) and fish oil (FO) and using a laboratory-scale spray–dryer with varying conditions: inlet air temperature (IAT, 125–185 °C), wall material (WM, 5–25%), pump speed (PS, 3–7 mL/min), and needle speed (NS, 3–11 s). The maximum alpha-linolenic acid (ALA) content was 33 ± 1%. The highest values of eicosapentaenoic acid (EPA) and docosahexaenoic acid (DHA) in the microcapsules were 8.4 ± 0.4 and 13 ± 1%, respectively. Fourier transform infrared and X-Ray diffraction analysis results indicated that SDM was successfully formulated with Gum Arabic and maltodextrin (MD). The blending without encapsulation of CSO and FO was digested more efficiently and resulted in more oil being released with simulated gastric fluid (SGF), simulated intestinal fluid (SIF), and SGF + SIF conditions without heating. No significant changes were observed for saturated, monounsaturated, and LCPUFA, whether exposed or not to gastrointestinal conditions. However, compared to the release of SDM, it can be useful for designing delivery systems for the controlled release of essential fatty acids.

## Introduction

The omega–3 (ω–3) and omega–6 (ω–6) fatty acids need to be balanced in the human diet. The importance of ω–3 enriched foods for health seems to be expanding^1,2^. The ω−3 and ω−6 fatty acids have different functional roles in humans. Humans do not synthesize ω–3 fatty acids despite their important physiological functions^3^. Therefore, they must be supplied in the diet. Different processes have been used to improve the amount of ω−3 in foods such as the addition of ω−3 to animal feed. This can increase the levels of ω−3 in meat, eggs, and milk. Additionally, some plants can synthesize both ω−3 and ω−6 fatty acids^4,5^.

The essential ω−3 polyunsaturated fatty acids (PUFA) can be derived from various traditional sources. Seeds and fish oil (FO) are recognized as good sources of ω−3 fatty acids. Chia seeds (CS) are a good source of PUFA, which is important for health. CS is an ancient crop from the southern part of North America. The seeds have been traditionally used as a food and as an herbal medicine. They are also being used for their functional properties^6–8^. In many research works, it has been indicated that different types of CS contain ~ 25–35% oil content^9–11^. Nadeem and Imran^12^ reported that the fatty acids composition of CS oil (CSO) mainly consists of the essential fatty acid alpha-linolenic acid (ALA) at ~ 60–65% of the total fatty acids in the sn-2 position of the tri-acylglycerol. The major ω−3 PUFA, namely eicosapentaenoic acid (EPA) and docosahexaenoic acid (DHA) are present in significant amounts in fish fillets^13^. The oil extracted from fish fillets of *Labeo rohita* contains a significant level of EPA (16–18%) and DHA (10–12%)^14^. Therefore, the blend of chia and fish oil may lead to an ω−3 enriched oil with beneficial health effects^15,16^. These long chain (LC) PUFA have been shown to have an important role in human nutrition. They also have curative and preventive effects with respect to numerous human diseases such as heart diseases, chronic cancers, skin disorders, rheumatoid arthritis, and chronic inflammation^17,18^.

These oil blends have potential health benefit applications in food, pharmaceutical, and cosmetic sectors for the production of ω−3 enriched products^19^. Lipid deterioration is one of the most common issues affecting fatty acid stability in oil via free-radical propagated chain reactions. LCPUFA in the blend of FO and CSO have reduced their stability due to different conditions of humidity, temperature, light, oxygen, and enzymatic rancidity (peroxidases and lipases)^20,21^. ALA is rapidly oxidized, followed by EPA and DHA during food processing & preservation and storage intervals^22^. The fatty acid stability of these oils can be improved using encapsulation with specific conditions^23^. There are many methods to encapsulate foods but spray–drying methods have been recognized as a the oldest and the most widely used to prepare spray–dried microcapsules (SDM) of all types of food ingredients like fat and oil, flavors, and bioactive components. Spray-drying technique is available, inexpensive, easy to process, and quicker^24,25^. Therefore, protecting ω−3 fatty acids in foods against oxidation would be beneficial. For the production of SDM, the four most important factors seem to be inlet air temperature (IAT), wall material (WM), pump speed (PS), and needle speed (NS)^26–28^. In the first phase, an emulsion is prepared. Gum Arabic (GA) and maltodextrin (MD) are often used as core materials currently being used in the pharmaceutical, nutraceutical, and food processing and preservation industries^14^. The oxidative degradation of ω–3 enrich oil after spray–drying is associated with the profile of the WM^16,29,30^. Fine particles are produced with diameters of ~ 1–5 µm. These are internally coated with a thin nano–thick antistatic film to improve the stability. These are then spray–dried to produce SDM^31–33^. The current objective is to spray–dry ω–3 enriched oils at optimum conditions and study their impact on the fatty acid profile and in vitro digestibility of the SDM.

## Materials and methods

### Procurement of raw materials

CS (Gazala’s Pantry, Faisalabad, Punjab, Pakistan) and skin-on fish fillets (*Labeo rohita,* rohu, a member of the carp family) were purchased from an SB Department Store, Faisalabad, Punjab, Pakistan). All Sigma–Aldrich^®^ chemicals (St. Louis, MO, USA) were procured from the scientific stores in Punjab, Pakistan, and were at least research grade. The seeds were cleaned by washing with tap water and then dried at room temperature (27 ± 2 °C) to remove any dirt and other unnecessary materials. Fillets used for oil extraction were ~ 1 m long and ~ 2 kg. A total of ~ 50 kg of fish fillets were used.

### Chemical composition of the raw materials

#### Moisture contents (MC)

The MC of the samples was estimated using the official AOAC method 930.15^34^. Samples (5 g) were dried in a hot-air-oven (Memmert, Äußere Rittersbacher, Germany) at 105 °C for 1 h in petri dishes (Fudau Cell Culture, Luolong, Henan, China). A digital scale (SF–400A, The Stationers, London, UK) was used for all weighing. After heating, the samples were cooled in a desiccator containing phosphorus pentoxide. The MC was calculated:

1 $$MC \left( \% \right) = \frac{{{\text{W}}2 - {\text{ W}}3}}{{{\text{W}}2 - {\text{W}}1}} \times 100$$

where: W1 = Weight of petri dish, W2 = Weight of petri dish with sample, W3 = Weight of petri dish with sample after drying, W2–W3 = Loss of moisture, W2–W1 = Weight of fresh sample.

#### Ash content

The ash content was estimated using the AACC^35^ basic method 08–01. A 5 g sample was poured into a dry, pre–weigh crucible. Samples were placed in an electric muffle furnace (FHX–12, Daihan Scientific, Largo, FL, USA) at ~ 500–550 °C for 6 h. The crucibles were cooled in a desiccator and weighed immediately after room temperature was attained. The ash content was:

2 $$Ash contents \left( \% \right) = \frac{{{\text{Wr}}}}{{{\text{Ws}}}} \times 100$$

where; Wr = Weight of residue, Ws = Weight of sample.

#### Crude protein

The crude protein was estimated using the oldest AOAC^34^ Kjeldahl method 64–50. A 2 g sample in a 10 mL test tube (Taian Youlyy, Xintai, Shandong, China) had a digestion tablet added (Merck, St. Louis, MO, USA) and 20 mL of 0.1 M H_2_SO_4_ for digestion. After 3–4 h digestion, a bright yellow color was obtained. The solution was cooled at room temperature. After cooling, distilled water was added to 50 mL. H_2_SO_4_ to trap the ammonia that was then volatilized during the distillation process. The ammonia was collected into a flask with 4% boric acid and a methyl indicator. The mixture was back titrated with 0.1 N H_2_SO_4_ to determine total nitrogen (Eq. 3). A conversion factor was used to convert nitrogen to crude protein. Crude protein (%) = Nitrogen % × 6.25, which was the calibration factor selected.

3 $$Nitrogen \left( \% \right) = \frac{{\left( {{\text{A}} - {\text{B}}} \right) \times {\text{N}} \times 1.4007}}{{{\text{W }}\left( {\text{g}} \right)}}$$

where: A = mL of alkali of blank, B = mL of alkali of sample, N = Normality of alkali, W = Weight of sample.

#### Crude fat

The fat was measured with minor modifications using the AACC^36^ official method 30–10. A 2 g sample of raw material was wrapped in filter paper (grade 40: 8 μm). Then, 2 mL of 95% ethanol was gently added. After that, 10 mL of HCl was added and gently stirred. The beaker was placed in a water bath at 75 °C and mixed for 0.5 h until the solution was completely hydrolyzed. Then, 25 mL of ether was added to the solution and stirred for 1 min. The rinsed contents of the beaker were added into an extraction tube with 25 mL of redistilled petroleum ether (Sigma Aldrich, St. Louis, MO, USA) and again agitated for 60 s. The final solution was kept at room temperature with no disturbance until a layer of fat appeared on the fluid surface. The fat content was then alienated from the solution using centrifugation (Megafuge 8R Small Benchtop Centrifuge, Thermo Fisher Scientific, Dreieich, Germany) at 30,300 × g (600 rpm with a fixed angel rotor) for 15–20 min. After centrifugation, the fat content was filtered through Whatman No. 1 filter paper (Cytiva, Marlborough, MA, USA). The solvents were volatilized in a hot air oven at 100 °C for half hour.

#### Crude *fiber*

Crude fiber was obtained using AOAC^34^ Method No. 978.10. Sample (3 g) was digested using boiling H_2_SO_4_ (1.25%). Distilled and filtered through Whatman No. 1, water was used to wash the sample from the beaker. Samples were re–digested using NaOH (1.25%) and the beaker re–washed as previously. Then, the filtrate sample was collected in a dried crucible and placed on a hot plate to remove the excess water. The crucible was placed in a hot air oven for 2 h at 230 °C. The residues of the sample in the crucible were ashed in the muffle furnace for 3–5 h at 550–650 °C. The amount of crude fiber was estimated according to Eq. (4).

4 $$Crude fiber \left( \% \right) = \frac{{{\text{W}}1 - {\text{W}}2}}{{{\text{Ws}}}} \times 100$$

Where: W1 = Weight of crucible with fiber, W2 = Weight of crucible with ash, Ws = Weight of sample.

#### Nitrogen free extracts (NFE)

The NFE was:

5 $$NFE \left( \% \right) = 100 - Mositure \left( \% \right) + Crude fat \left( \% \right) + Crude fiber \left( \% \right) + Ash \left( \% \right)$$

### Oil extraction

CSO and FO were extracted using a cold press extraction and solvent extraction, respectively, according to the procedure of Rahim et al.^21^. Briefly, the CSO was extracted using a mini oil press model 6YL-550. The undesirable materials were removed from the extracted oil by sedimentation at room temperature for 24 h. The extracted oil was filtered through Whatman No. 1 and stored in the dark in screw-capped clear plastic bottles for a maximum of 1 wk. For the FO, solvent mixtures were prepared using methanol (Sigma Aldrich, 100 mL) and chloroform (Sigma Aldrich, 50 mL) at 27 ± 2 °C. Each fish sample (100 g) was soaked for 12 h in the already prepared solvent mixtures. A rotary evaporator was further used to evaporate the solvent mixture at 50 °C. The FO was saved in the dark in the plastic bottles for a maximum of 1 week.

### Emulsion preparation

A blend of CSO and FO (50:50%) was produced at room temperature. All emulsions were prepared in the same manner as described in the recent research work of Rahim et al.^14^. An emulsion was progressively formed by combining 97 mL of distilled water with 3 mL of toluene. A 15 mL blend of CSO and FO and 85 mL of the above solution were gently mixed in another beaker. Then, soy lecithin (1%, w/w) was utilized as a natural emulsifier and stabilizer. Moreover, GA and MD (1:1 mass ratio, 15 g) were added as a wall material (WM) with a magnetic stirrer for 15 min. A homogenizer was used to mix at 110,00 rpm for 10 min.

#### Spray–drying

SDM were prepared in a lab–scale mini spray–drier (Model number TPS–15, Toption Co., Shanghai, China)^37^. The spray–drier operating conditions for optimization were IAT of 125, 140, 155, 170, and 185 °C; WM of 5, 10, 15, 20, and 25%; PS of 3, 4, 5, 6, and 7 mL/min; and NS of 3, 5, 7, 9, and 11 s. The spray–dryer was preheated to the required temperature before feeding. A hydrostatic pump was used to pump the homogenized samples into the atomizer at the required flow rate. Then, the atomizer sprayed the raw materials into the drying chamber with hot air. Powder and gas entered into a cyclone separator, where they were separated in a glass collection tube. The SDM were inserted in a polythene zip bags (Local market, Faisalabad, Punjab, Pakistan) and kept at 27 ± 2 °C and humidity 40 ± 45% for a maximum of three weeks.

### Characteristics of SDM

#### Fatty acid composition

Using the process of Rahim et al.^21^, methyl esters of fatty acids were evaluated using a gas chromatograph (GC, Model 7890–B, Agilent Technologies, Santa Clara, CA, USA). The esters of the samples were prepared using the standard method Ce 1f.–96 with some changes as explained by AOCS^38^. Briefly, 2 g of samples were inserted into a glass test tube with 2 mL of 1 M of caustic soda in methanol (100% concentration). Then, test tubes were placed into a boiling water bath at 95 °C until the mixture of samples became colorless. Then 3 mL of boron trifluoride was gently mixed-in and re-heated at 95 °C for 10 ± 3 min. After heating, hexane (3 mL) was mixed vigorously until two phases appeared. The supernatant was poured into GC vials and the peak areas were calculated using the software with the instrument and assumed to represent percent concentration assuming an equal response to each peak on a weight basis. The gas flow velocity for He, H_2_, and O_2_ (ratio 2:4:40) was adjusted from 20 to 25 mL/min at 185 °C to estimate the fatty acid profile using a SP–2560 capillary column 100 m × 0.25 mm id (Agilent Technologies, model 7890 B). FAME 37 (Supelco, St. Louis, MO, USA, a C37 alkane) and SLB–IL111 (Merck, St. Louis, MO, USA) internal standards were used for the fatty acid profile and their isomers.

#### Fourier transform infrared (FTIR) and X-Ray diffraction analysis (XRD)

FTIR was operated from ~ 650 to 3500 cm^−1^ using an Agilent Technologies Cary 630 to assess the quantitative and qualitative analysis of the samples. The oil blend sample (S_1_), wall material sample (S_2_), and SDM sample (S_3_) were placed onto flat glass plates. Further, a very clean another plate was placed direct on top to get a clean film. The plate was kept in the special sample holder and the spectral wavenumber resolution was run at 4 cm^−1^ at ambient temperature until the CO_2_ peaks were minimized. Agilent MicroLab software was used to evaluate the signals and identify the peaks^39^. The crystallinity of samples was performed using an X-ray diffractometer (Model D8, Bruker, Berlin, Germany). The samples were placed in a slot and then pressed with frosted glass to get a good texture. XRD patterns were recorded at room temperature with a wavelength of 1.54 Å, angle 2θ ranging from 25 to 80º, and the instrument operated at 30 kV.

### Impact of SDM in vitro with a simulated gastro–intestinal environment

#### Preparation of simulated gastric fluid (SGF)

A SGF was used for the in vitro release behavior of SDM as described by Minekus et al.^40^ with minor changes. NaCl (2 g) and 7 mL HCl (36%) were mixed with 900 mL deionized water (Sigma Aldrich) using a magnetic stirrer for 5 min and 3.2 g pepsin (Sigma Aldrich) to obtain a pH of 2.0 and the solution brought to 1000 mL with deionized water. The solution was incubated at 37 °C for 120 min with mixing at 100 rpm in an incubator shaker and kept at 4 ± 0.5 °C in a refrigerator for a maximum of 3 days.

#### Preparation of simulated intestinal fluid (SIF)

A SIF was prepared according to Goyal et al.^41^. A stock solution was prepared by dissolving 6.8 g of KH_2_PO_4_ in 850 mL of distilled water. The 0.2 M NaOH and 100 g of 1 × USP pancreatin (Sigma Aldrich) were brought to pH 1.2 with the hydrochloric acid solution, 0.1 M. The solution was incubated in the dark with mixing at 4 °C for 6 h and brought to 1000 mL with distilled water. It was refrigerated for a maximum of 3 days.

#### In vitro release behavior of SDM with SGF

The samples were prepared in test tubes by adding 5 g SDM containing 50% oil (w/w basis) to 900 mL of SGF. These were kept in the dark at 38 °C for 2 h. After incubation, 30 mL of petroleum ether and diethyl ether at 1:1 were added with magnetic stirring. A separatory funnel was used to separate the oil from the solution at room temperature. The separated oil was heated at 80 °C until the solvent evaporated. The oil was dried in the hot air oven for 30 min at 100 ± 5 °C^42^. The amount of oil in the capsule was calculated using the amount of starting oil in the SDM (loading capacity) and the percentage of oil released in each synthetic digestive fluid:

6 $$Oil release \left( \% \right) = \frac{{{\text{Ao}}}}{{{\text{As}}}} \times 100$$

where, Ao = Amount of oil release, As = Amount of sample.

#### In vitro release behavior of SDM with SGF and SIF

To determine the oil release from SDM, 60 mL of both SGF and SIF were put in separate 500 mL beakers and mixed with 6 mL SDM. The SGF was incubated at 37 °C for 2, 3, and 4 h while shaking at 110 rpm. Subsequently, it was transferred into SIF and placed in the dark at 37 °C for 2 h and analyzed at 230 nm using a spectrophotometer (Analytik Jena AG–Specord 200 Plus, Jena, Germany). The quantity of released oil was calculated by evaporating the petroleum ether in a hot air oven at 80 °C for 30 min^43^.

### Model fitting and statistical analysis

A central composite design (CCD) of the response surface methodology (RSM) with a total of 30 runs was used to optimize the four independent factors, while the response variables were: ALA, EPA, and DHA. All spray–drying runs were mixed to reduce repeatable errors in the model. The optimal values were evaluated for their level of significance with 5% (*p* ≤ 0.05) being significant using the Stat–Ease® software (version 11.1.2.0, Minneapolis, MN, USA). A statistical technique analysis of variance (ANOVA) was assessed to calculate the significant differences between independent variables and the validity of the experimental design. The validity or adequacy of the CCD was clarified using an estimation of the regression coefficient, R_2_–adj, and the lack of fit. The operating conditions of the spray–drier were presented in coded and actual levels that were predicted using the CCD, the maximum level was + 2, while the minimum level was − 2. Six central points (C_1_–C_6_) were evaluated to determine the response error. The linearity of the CCD for the independent factors were evaluated for the different dependent factors to estimate the quadratic effect of the spray–drier operating variables. The inter–day repeatability was calculated by analysis of the same emulsion for spray–drying one time/day for four days^44^. The dependent variables obtained according to the CCD were fitted to a second–order polynomial model and regression coefficients calculated. Statistix (version 8.1, analytical software, Tallahassee, Florida, USA) was used for the in vitro analysis.

## Results and discussion

### Proximate analysis of raw material

The proximate analysis of the raw material samples is shown in Table 1. Imran et al.^45^ showed that the CS they studied contained 6.1% moisture, 4.1% crude ash, and 18.2% crude protein. Mehta and Nayak^46^ showed that the moisture of rohu fillets (*Labeo rohita*) was 81.4%. Gandotra et al.^47^ reported that the ash of rohu fillets at refrigerated temperature was 1.7% and the lipid was 3.8%. Mahboob et al.^48^ showed that *rohu* fish fillets contained 71.6–77.5% moisture, 16.6–30.6% crude protein and 1.6–4.6% NFE.

**Table 1:** Chemical composition of raw materials.

Parameter	Chia seeds (CS)	Rohu fish fillet
	Quantity (%)	
Moisture	5.9 ± 0.2^b^	78 ± 3^a^
Ash	3.3 ± 0.1^a^	2.4 ± 0.1^a^
Crude protein	17.4 ± 0.3^a^	12.6 ± 0.2^b^
Crude fat	32 ± 2^a^	3.1 ± 0.1^b^
Crude fiber	22.6 ± 0.4^a^	–
Nitrogen free extract (NFE)	16.7 ± 0.3^a^	3.6 ± 0.1^b^

Results are represented as mean ± standard deviation.

^a-b^Indicate different letters at the level of significance (*p* ≤ 0.05) in the same row.

### Optimization of SDM

#### Fatty acid composition

**Figure 1:**
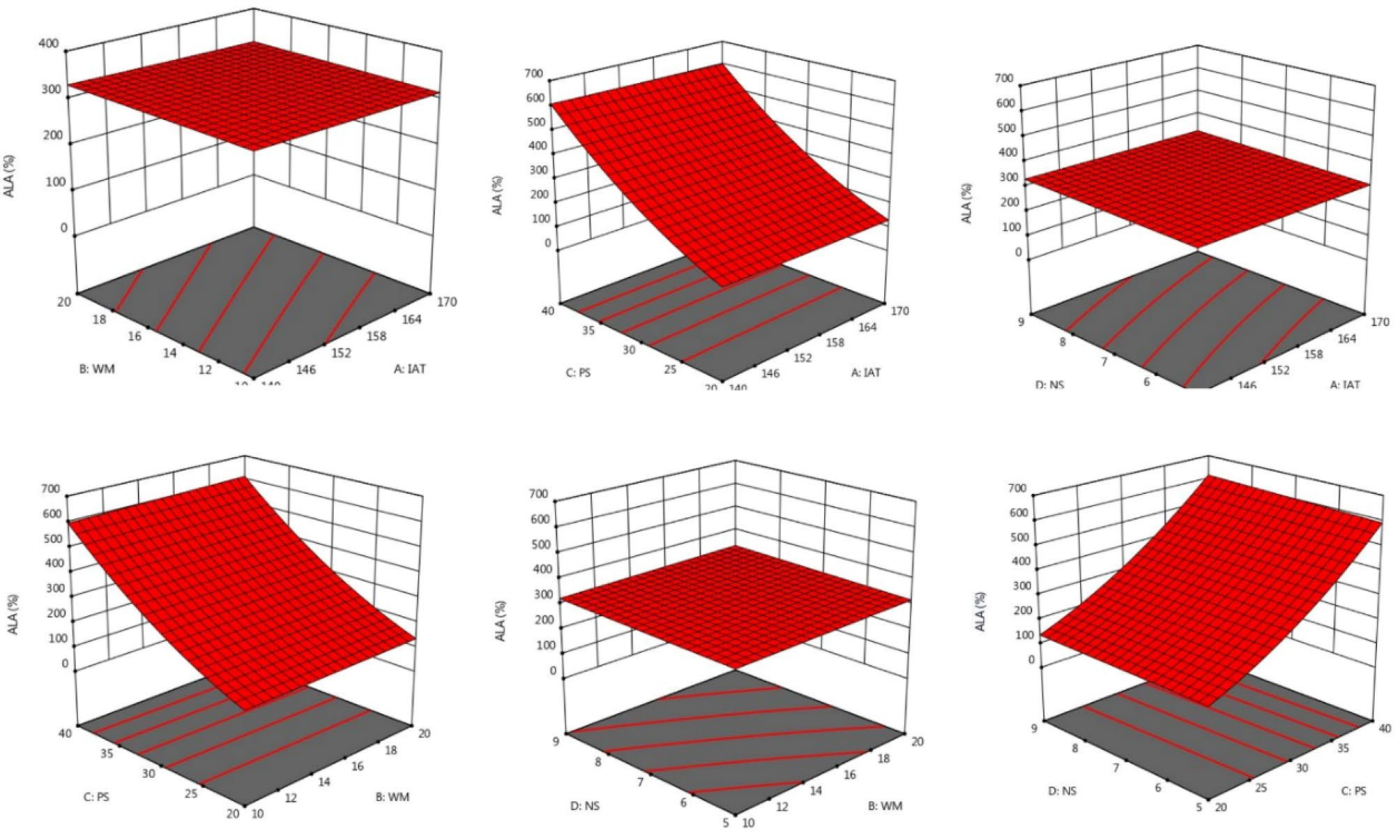
Three dimensional surface plots of mutual interaction impact of spray dryer operating variables on the alpha-linolenic acid (ALA).

**Figure 2:**
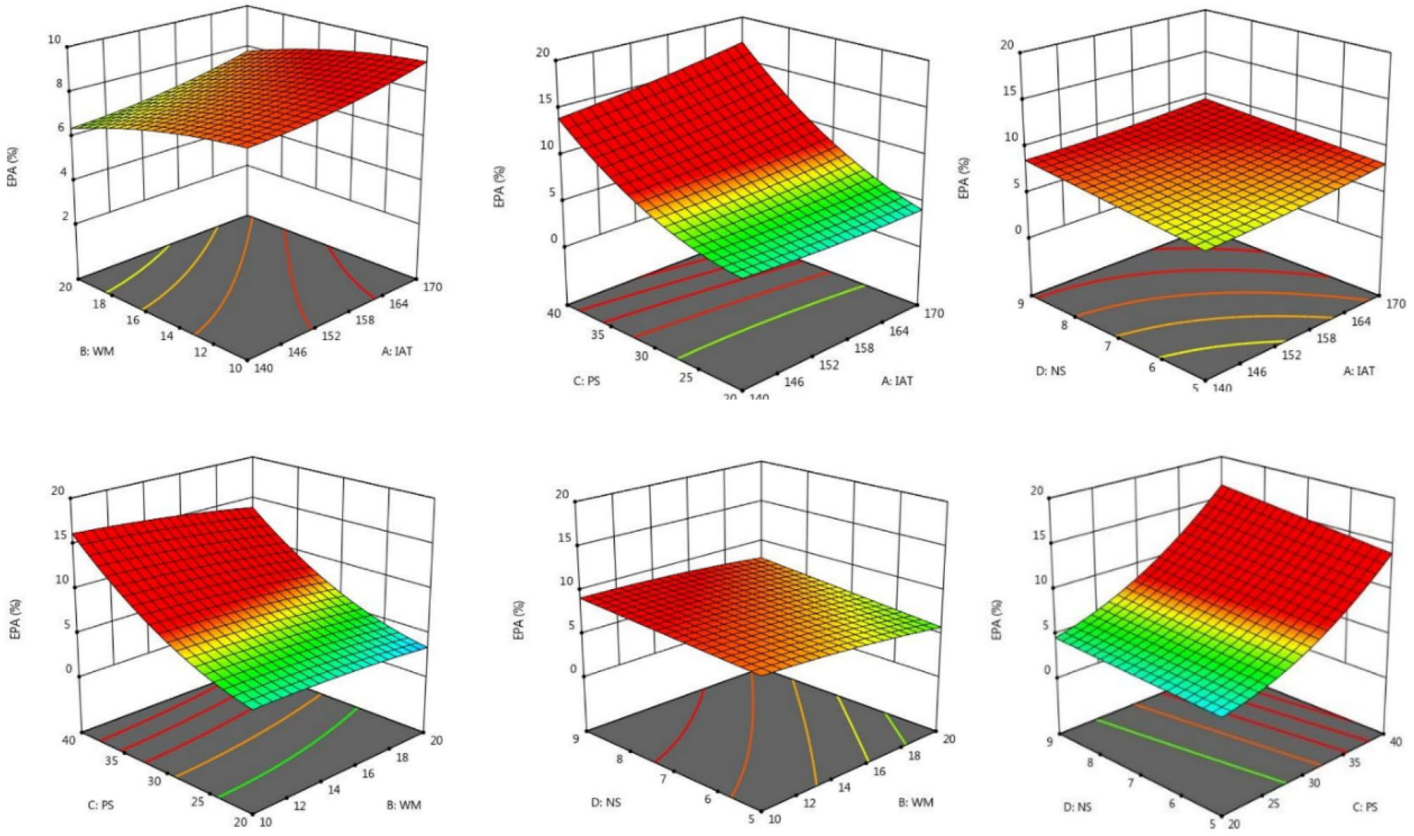
The interaction impact between spray-drying factors on eicosapentaenoic acid (EPA).

**Figure 3:**
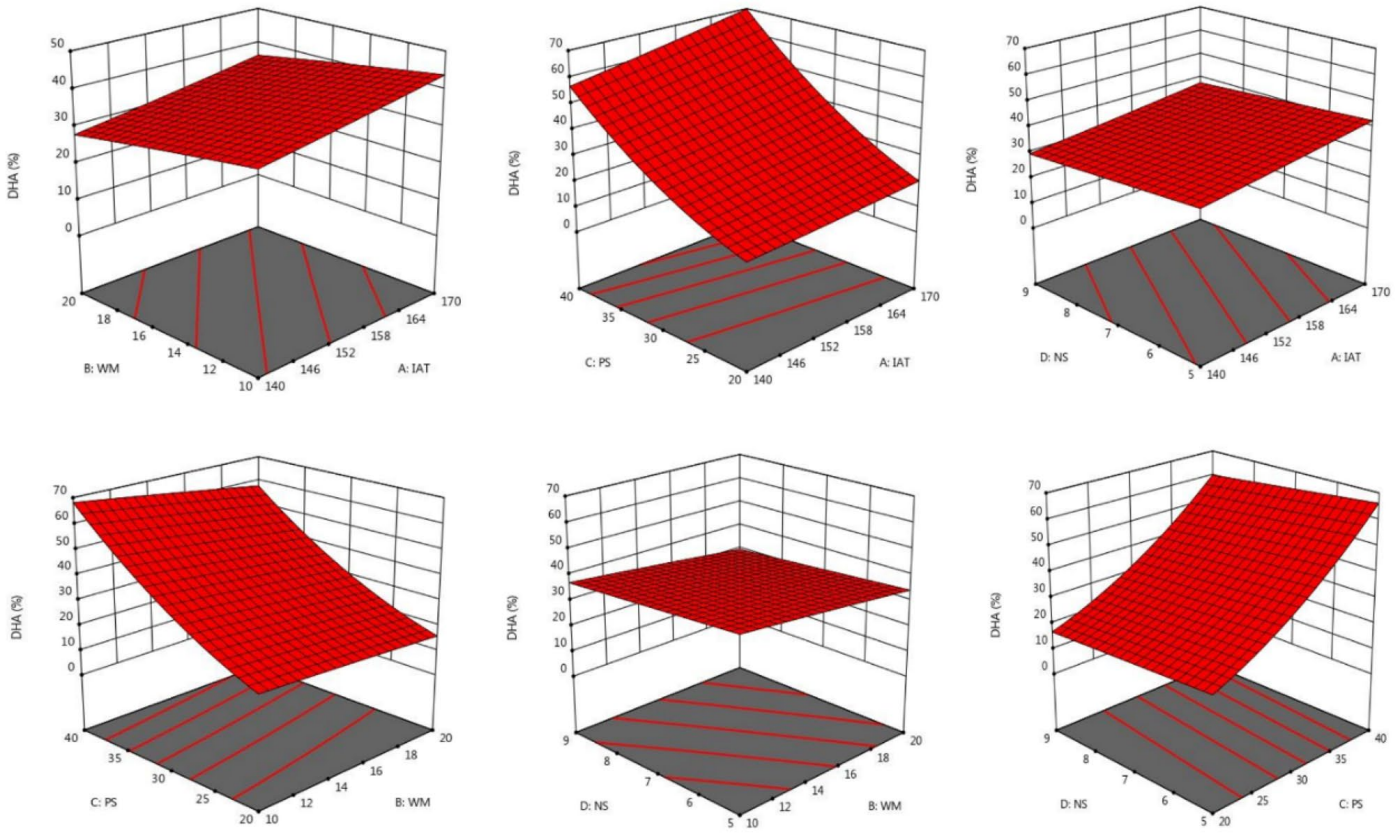
Effect of spray-drying variables on docosahexaenoic acid (DHA) of spray–dried microcapsules (SDM).

The SDM was optimized using a central composite design (CCD) using ALA, EPA, and DHA retention as % of total fatty acids as the dependent variables. The combined effects of the spray-drying variables on the fatty acid composition of SDM are described in Table 2. The maximum ALA was observed at IAT 140 (− 1); WM 10 (− 1); PS 4 (− 1); and NS 5 (− 1). The highest concentrations of EPA and DHA in SDM were for spray-drying run 9, and the lowest values were for run 26. The mutual interaction effect of WM and NS was non-significant and IAT and PS had the most significant effect on fatty acid composition as described in Figs. 1, 2 and 3. The optimized predicted value of ALA was directly proportional to the independent variables. The final data suggested that independent variables did not significantly impact the fatty acid profile of SDM (*p* > 0.05). The percentage of LCPUFA was related to the natural oil. ANOVA showed that the models were significant (*p* ≤ 0.05) for ALA retention, and the F value of lack of fit was also significant. ANOVA also indicated that the models were significant for EPA retention (*p* ≤ 0.05) and the lack of fit was also significant. The ANOVA for DHA retention indicated that the model was significant but the lack of fit was not significant (*p* > 0.05, Table 3). The linear regression equations were intended to observe the best regression fit model into the data. In this model for ALA, EPA, and DHA retention, the R^2^ value ranged from 0.84 to 0.93, with a tiny fraction of these dependent variables led to an Adjusted-R^2^ that increased from 0.69 to 0.87 indicating a better model. A lower Adjusted-R^2^ indicated that the independent variables were not adding value to the model. The predicted R^2^ ranged between 0.21 and 0.64, which was significant and the actual regression values are shown in Table 4. The fatty acid composition of SDM is altered by the presence of non–triglyceride components, heat, and fatty acid double bonds^49^. The fatty acid composition was consistent with Fernandes et al.^50^. The fatty acid composition of CSO and CS was retained at high-temperature (120 °C) spray–drying. Rahim et al.^21^ showed that the fatty acid losses in SDM of CSO and FO were in the acceptable range at 170 °C. The results were consistent with Lavanya et al.^15^; Ali et al.^51^ and Rahim et al.^39^, who established that there was no significant (*p* > 0.05) effect of spray dryer operating conditions on the fatty acid composition.

**Table 2:** CCD of independent variables for fatty acid profile of SDM.

Spray–drying run	Spray–drying operating factors				Response factors		
	IAT (°C)	WM (%)	PS (mL/min)	NS (S)	ALA (%)	EPA (%)	DHA (%)
1 (C_1_)	155 (0)	15 (0)	5 (0)	7 (0)	28 ± 1^d^	5.1 ± 0.2^kl^	9.9 ± 0.4^gh^
2 (C_2_)	155 (0)	15 (0)	5 (0)	7 (0)	27 ± 1^d^	5.2 ± 0.2^kl^	9.8 ± 0.4^gh^
3	155 (0)	15 (0)	5 (0)	3 (– 2)	28 ± 1^c^	5.8 ± 0.2^k^	10.9 ± 0.5^cd^
4	155 (0)	15 (0)	7 (+ 2)	7 (0)	32 ± 1^d^	4.6 ± 0.2^lm^	10.4 ± 0.5f.
5	140 (– 1)	20 (+ 1)	4 (– 1)	9 (+ 1)	30 ± 1^b^	7.8 ± 0.3^hi^	12 ± 1^c^
6	155 (0)	25 (+ 2)	5 (0)	7 (0)	28 ± 1^cd^	2.8 ± 0.1^ik^	9.1 ± 0.5^ef^
7	170 (+ 1)	10 (– 1)	4 (– 1)	5(– 1)	26 ± 1^de^	3.6 ± 0.1^mn^	7.6 ± 0.3^ k^
8 (C_3_)	155 (0)	15 (0)	5 (0)	7 (0)	27 ± 1^d^	5.1 ± 0.2^kl^	10.1 ± 0.5^gh^
9	140 (– 1)	10 (– 1)	4 (– 1)	5(– 1)	33 ± 1^a^	8.4 ± 0.4^hi^	13 ± 1^a^
10	170 (+ 1)	20 (+ 1)	4 (– 1)	5(– 1)	26 ± 1^de^	4.0 ± 0.2^m^	8.7 ± 0.4^h^
11 (C_4_)	155 (0)	15 (0)	5 (0)	7 (0)	27 ± 1^d^	5.2 ± 0.2^k^	9.9 ± 0.4^gh^
12 (C_5_)	155 (0)	15 (0)	5 (0)	7 (0)	27 ± 1^d^	5.1 ± 0.2^kl^	10.0 ± 0.5^gh^
13	155 (0)	5 (– 2)	5 (0)	7 (0)	28 ± 1^d^	4.9 ± 0.2^l^	9.6 ± 0.4^h^
14	140 (– 1)	10 (– 1)	6 (+ 1)	9 (+ 1)	30 ± 1^bc^	6.3 ± 0.3^i^	11.1 ± 0.5^e^
15	170 (+ 1)	10 (– 1)	6 (+ 1)	5(– 1)	24 ± 1^e^	3.3 ± 0.1^n^	9.0 ± 0.4^b^
16	170 (+ 1)	10 (– 1)	6 (+ 1)	9 (+ 1)	25 ± 1^e^	2.9 ± 0.1^no^	7.1 ± 0.3^kl^
17	170 (+ 1)	10 (– 1)	4 (– 1)	9 (+ 1)	26 ± 1^e^	2.6 ± 0.1^n^	7.4 ± 0.3^k^
18	140 (– 1)	20 (+ 1)	4 (– 1)	5(– 1)	32 ± 1^ab^	7.1 ± 0.3^hi^	13 ± 1^ab^
19	140 (– 1)	10 (– 1)	4 (– 1)	9 (+ 1)	30 ± 1^bc^	6.8 ± 0.3^hi^	12 ± 1^cd^
20	140 (– 1)	10 (– 1)	6 (+ 1)	5(– 1)	29 ± 1^b^	6.1 ± 0.3^i^	11.8 ± 0.5^d^
21	185 (+ 2)	15 (0)	5 (0)	7 (0)	29 ± 1^e^	3.1 ± 0.1^mn^	8.4 ± 0.4^l^
22	140 (– 1)	20 (+ 1)	6 (+ 1)	9 (+ 1)	29 ± 1^c^	7.4 ± 0.3^hi^	11.4 ± 0.5^de^
23 (C_6_)	155 (0)	15 (0)	5 (0)	7 (0)	27 ± 1^d^	5.2 ± 0.2^kl^	9.9 ± 0.4^gh^
24	155 (0)	15 (0)	5 (0)	11 (+ 2)	28 ± 1^d^	4.2 ± 0.2^l^	9.4 ± 0.4^hk^
25	155 (0)	15 (0)	3 (– 2)	7 (0)	28 ± 1^c^	5.5 ± 0.3^ik^	10.1 ± 0.5^fg^
26	170 (+ 1)	20 (+ 1)	6 (+ 1)	9 (+ 1)	21.5 ± 0.5^e^	2.2 ± 0.1^p^	6.9 ± 0.3^m^
27	170 (+ 1)	20 (+ 1)	4 (– 1)	9 (+ 1)	22 ± 1^e^	4.3 ± 0.1^lm^	7.9 ± 0.4^k^
28	170 (+ 1)	20 (+ 1)	6 (+ 1)	5(– 1)	24 ± 1^e^	3.8 ± 0.2^ m^	8.3 ± 0.4^hk^
29	125 (– 2)	15 (0)	5 (0)	7 (0)	30 ± 1^ab^	8.6 ± 0.3^i^	13 ± 1^c^
30	140 (– 1)	20 (+ 1)	6 (+ 1)	5(– 1)	32 ± 1^ab^	6.5 ± 0.3^i^	11.6 ± 0.5^d^

*SDM* Spray–dried microcapsules, *IAT* Inlet air temperature, *WM* Wall material, *PS* Pump speed, *NS* Needle speed, *ALA* Alpha-linolenic acid, *EPA* Eicosapentaenoic acid, *DHA* Docosahexaenoic acid, *C1-C6* Represents the central points of the central composite design.

^a-gh^Indicate different letters at the level of significance (*p* ≤ 0.05) in the same column.

**Table 3:** ANOVA for spray–drying operating factors’ impact on the response variables for SDM.

Source of variation		DF	ALA (%)		EPA (%)		DHA (%)	
			MS	*p–value*	MS	*p–value*	MS	*p–value*
Model		14	0.25*	< 0.0001	0.12*	0.0001	1.00*	0.0009
Linear effects	IAT	1	0.12*	0.88	0.004^N.S^	0.61	0.24*	0.25
	WM	1	0.004^N.S^	0.59	0.0006^N.S^	0.84	0.35*	0.17
	PS	1	0.01^N.S^	0.42	0.0002^N.S^	0.90	0.51*	0.10
	NS	1	4.004**	0.61	0.0002^N.S^	0.92	0.08^N.S^	0.11
Interaction effects	IAT × WM	1	0.12*	0.48	0.11*	0.59	0.17*	0.33
	IAT × PS	1	0.001^N.S^	0.75	0.0005^N.S^	0.85	0.05^N.S^	0.17
	IAT × NS	1	0.01^N.S^	0.39	0.0008^N.S^	0.82	0.24*	0.25
	WM × PS	1	0.004^N.S^	0.58	0.0003^N.S^	0.89	0.35*	0.17
	WM × NS	1	0.002^N.S^	0.69	0.0002^N.S^	0.92	0.08^N.S^	0.48
	PS × NS	1	1.21*	0.53	2.23**	0.95	0.42*	0.14
Quadratic effects	IAT^2^	1	0.09^N.S^	0.03	0.12*	0.69	0.14*	0.38
	WM^2^	1	0.003^N.S^	0.64	0.001^N.S^	0.74	0.05^N.S^	0.59
	PS^2^	1	0.01^N.S^	0.38	0.0005^N.S^	0.86	0.52*	0.10
	NS^2^	1	1.02*	0.21	0.003^N.S^	0.65	0.49*	0.11
Residual	15	10.01**	–	0.01^N.S^	–	2.06*	–	
Lack of fit		10	16.02**	0.24	0.02^N.S^	0.01	1.09*	0.21
Pure error		5	0.74^N.S^	–	0.002^N.S^	–	0.002^N.S^	–
Cor total		29	–	–	–	–	–	–

*SDM* Spray–dried microcapsules, *ALA* Alpha-linolenic acid, *EPA* Eicosapentaenoic acid, *DHA* Docosahexaenoic acid, *DF* Degree of freedom, *MS* Mean squares,* Significant, *N.S* Non-significant, *IAT* Inlet air temperature, *WM* Wall material, *PS* Pump speed, *NS* Needle speed.

**Table 4:** Coded and actual regression equations for response factors after spray–drying.

Dependent variables	Regression form	Regression equations
ALA (%)	Coded	$$\begin{aligned} R1 & = + 40.99 - 0.11{\text{A}} - 0.43{\text{B + 10}}{\text{.02C + 0}}{\text{.41D}} - 0.02{\text{AB + 0}}{\text{.10AC + 0}}{\text{.02AD}} \\ \quad - 0.17{\text{BC}} - 0.01{\text{BD + 0}}{\text{.20CD + 0}}{\text{.05A}}^{2} + 0.01{\text{B}}^{2} + 2.15{\text{C}}^{2} + 0.03{\text{D}}^{2} \\ \end{aligned}$$
	Actual	$$\begin{aligned} R2 & = + 41.71 - 0.08{\text{IAT}} + 0.05{\text{WM}} - 0.40{\text{PS}} - 0.32{\text{NS}} - 0.00030{\text{IAT*WM}} + 0.0006{\text{IAT*PS}} + 0.00091{\text{IAT*NS}} \\ & \quad - 0.003{\text{WM*PS}} - 0.001{\text{WM*NS}} + 0.01{\text{PS*NS}} + 0.0002{\text{IAT}}^{2} + 0.0004{\text{WM}}^{2} + 0.02{\text{PS}}^{2} + 0.007{\text{NS}}^{2} \\ \end{aligned}$$
EPA (%)	Coded	$$\begin{aligned} R3 = & + 3.69 - 0.39{\text{A}} + 0.15{\text{B}} + 1.42{\text{C}} - 0.07{\text{D}} + 0.01{\text{AB}} - 0.05{\text{AC}} - 0.006{\text{AD}} \\ & \quad + 0.04{\text{BC}} + 0.003{\text{BD}} - 0.01{\text{CD}} - 0.009{\text{A}}^{2} + 0.007{\text{B}}^{2} + 0.40{\text{C}}^{2} - 0.01{\text{D}}^{2} \\ \end{aligned}$$
	Actual	$$\begin{aligned} R4 & = + 4.689 - 0.003{\text{IAT}} - 0.04{\text{WM}} - 0.05{\text{PS}} + 0.05{\text{NS}} + 0.02{\text{IAT*WM}} - 0.0003{\text{IAT*PS}} - 0.0002{\text{IAT*NS}} \\ & \quad + 0.0008{\text{WM*PS}} + 0.0003{\text{WM*NS}} - 0.0009{\text{PS*NS}} - 0.04{\text{IAT}}^{2} + 0.0003{\text{WM}}^{2} + 0.0040{\text{PS}}^{2} - 0.002{\text{NS}}^{2} \\ \end{aligned}$$
DHA (%)	Coded	$$\begin{aligned} R5 = & + 94.07 + 3.10{\text{A}} - 3.70{\text{B}} + 68.75{\text{C}} - 4.35{\text{D}} - 0.10{\text{AB}} + 1.49{\text{AC}} - 0.12{\text{AD}} \\ & \quad - 1.49{\text{BC}} + 0.07{\text{BD}} - 1.63{\text{CD}} + 0.07{\text{A}}^{2} + 0.04{\text{B}}^{2} + 13.80{\text{C}}^{2} - 0.13{\text{D}}^{2} \\ \end{aligned}$$
	Actual	$$\begin{aligned} R6 & = + 24.76 - 0.14{\text{IAT}} + 0.27{\text{WM}} - 1.93{\text{PS}} + 0.33{\text{NS}} - 0.001{\text{IAT*WM}} + 0.009{\text{IAT*PS}} - 0.004{\text{IAT*NS}} \\ & \quad - 0.02{\text{WM*PS}} + 0.007{\text{WM*NS}} - 0.081{\text{PS*NS}} + 0.0003{\text{IAT}}^{2} + 0.001{\text{WM}}^{2} + 0.13{\text{PS}}^{2} + 0.03{\text{NS}}^{2} \\ \end{aligned}$$

*ALA* Alpha-linolenic acid, *EPA* Eicosapentaenoic acid, *DHA* Docosahexaenoic acid, *R1*, *R2*, *R3*, *R4*, *R5*, *R6*, *R7*, *R8* Response factors, *A* Inlet air temperature (IAT), *B* Wall material (WM), *C* Pump speed (PS), *D* Needle speed (NS).

### FTIR analysis

**Figure 4:**
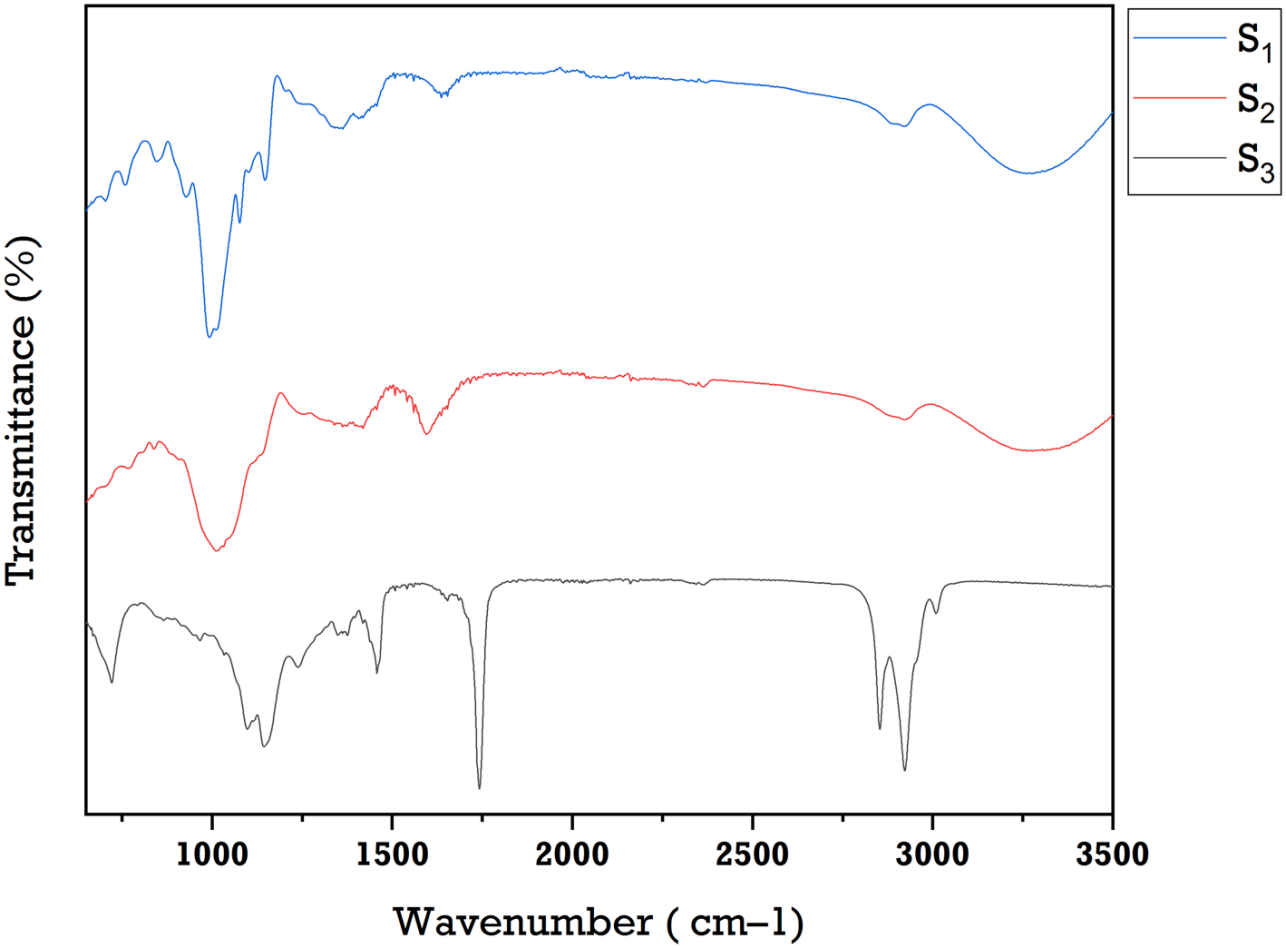
FTIR analysis of oil blend sample (S_1_), wall material sample (S_2_), and SDM sample (S_3_).

As can be seen in Fig. 4, there are several peaks in the FTIR of analysis of the oil blend sample (S_1_), wall material sample (S_2_), and SDM sample (S_3_) obtained at run order 9 that can be used for qualitative and quantitative analysis of functional components. In S_1_, the highest peak was observed at 3009 cm^−1^ while the lowest peak was experiential at 721 cm^−1^. The regions of 3009, 2922, and 2853 cm^−1^ were related to unsaturated systems and long–chain linear aliphatic compounds. Based on the infrared absorption peak value of the characteristic peaks and the intensity of its own C=O stretching group of the triglyceride peak (wave number: 1747 cm^−1^). Meanwhile, FTIR bands at 1653, 1459, 1375, and 1349 cm^−1^ represent the bending vibration of C=C stretching (α, β—unsaturated ketone), while those at 1239–721 cm^−1^ represent the stretching of C=O ester group. In S_2_, the peaks in the range of 3278–2922 cm^−1^ of an FTIR spectrum represent the C−H stretching vibration of the C–H link adjoining the double C–C link of lipids. The peaks in the range from 1992 to 2363 cm^−1^ represent the organic functional group, while the 1595–1362 cm^−1^ peak area represents the C=C stretching and C–H bending or aromatic rings of lipids. A sharp peak at 1013 cm^−1^ represents the absorption of the C−O bond from the ester in the triacylglycerol. In S_3_, the peaks at 1718, 1653, 1636, 1407, 1362, 1146, 1101, and 1075 cm^−1^ characterize carbonyls. A sharp peak at 993 cm^−1^ was assignable to the C=C bending of the alkene group. Peaks at 848, 704, and 758 cm^−1^ indicate the presence of C–H deformation. According to Bordon et al.^30^, a major absorption band at 3000 cm^−1^ shows the presence of ALA in microcapsules of CS. Lavanya et al.^15^ found an intensive peak at 2922 cm^−1^ showing the presence of LCPUFA in spray-dried rohu fatty acids. Karaaslan et al.^52^ have reported similar absorption at 3285 cm^−1^ in the case of GA/MD.

#### XRD

**Figure 5:**
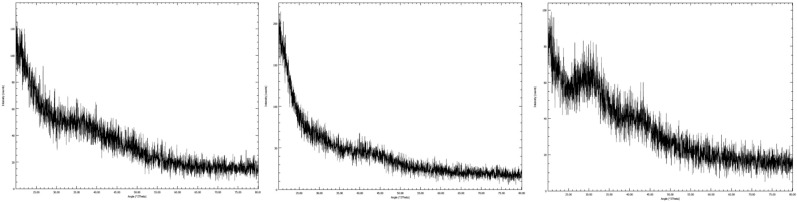
Shows the X-Ray diffraction analysis (XRD) of the oil blend sample (S_1_), wall material sample (S_2_), and SDM sample (S_3_).

The XRD spectra of S_1_, S_2_, and S_3_ are shown in Fig. 5. The sharp peak of S_1_ was obtained at 30°, which indicated the crystalline. S_2_ displayed no obvious characteristic peak, showing that the excipient itself was in an amorphous solid. Moreover, the XRD profile of S_3_ revealed broader peaks at 2θ angles of 30.1, 40.2, 43.3, and 53.17°, which indicated microcapsules prepared using wall materials had an amorphous nature with broader peaks. The results indicated that SDM was more soluble in water and less likely to release the wall material, SDM would be more ideal for enrichment or fortification. The XRD peaks also showed the successful microencapsulation of the chia/fish oil blend within the GA and MD. Similar data were obtained by Loughrill et al.^53^ the spray-dried powder of fish oil exhibited an amorphous structure. Another research work carried out by Sukumar et al.^54^ also indicated an amorphous XRD pattern in spray-dried chia seed oil.

### In vitro study of SDM digestion in SGF and SIF

Many encapsulation methods not only protect the LCPUFA oils against lipid peroxidation but also improve the behavior and functionality of ω–3–enriched oils in various food products. The main role of the WM in spray–drying is to simulate the drying of the core material by improving the crust on SDM. These WM are commercially used to prepare ω–3–enriched SDM in fortified food products because they are easy to use, low cost, and have good emulsifying properties. In addition to prolonging the oxidative stability of oils, the profile of the released active ingredients from encapsulation in the human gastrointestinal tract is important for determining the bioavailability of encapsulated oils^41,55,56^. MD is considered as the best encapsulating WM and helps control the release of oil during in vitro digestions^57^. Spray–drying is assumed to be the best technique for preserving these LCPUFA in oils according to Laohasongkram et al.^58^. Burgar et al.^59^ studied the characteristics of microencapsulated FO and estimated the behavior of oil and microcapsules in vitro. Similarly, SDM of CSO was developed using WM such as MD and sodium caseinate. The results showed that microcapsules that contained more sodium caseinate as WM showed the highest oil release rates with SIF with maximum loading capacity^60^.

The in vitro digestion results are shown in Table 5. The data indicated that the blending/non–encapsulation of CSO and FO was efficiently digested with SGF, SIF, and SGF + SIF without heat. However, the release of oil (RO) of SDM went from 42 ± 2% without heating to 63 ± 3% with heating. Furthermore, there was a slight increase in the RO for both CSO and FO blend/un–encapsulated and SDM with 2–4 h incubation without heating (Table 6). In a typical digestion process, food first is subject to gastric conditions followed by intestinal conditions. During this whole digestion process, there is a shift in pH and enzymes from gastric to intestinal fluids. Therefore, oil release was observed with both SGF and SGF + SIF. The quantity of RO with SIF was due to the formation of a compact surface that remained unaffected with SGF or it could be due to the protein-to-protein linkages and accumulation of proteins within a high acidic environment in the gastric fluids, which has been considered normal in many studies^61^. The in vitro releases were consistent with Binsi et al.^62^. They used GA as the WM with different ratios for spray–dried rohu FO. Their RO release was 96% in the case of stabilized powder and 88% for powder samples not stabilized prior to SGF. On the other hand, the oil released by SIF was 34% with un-stabilized powder and 32% with stabilized powder. The results for in vitro release are consistent with Binsi et al.^63^. They used GA as the WM at different ratios for spray-drying rohu FO. Their RO was 72% with SGF and 15% with SIF. Timilsena et al.^43^ studied the RO from spray–dried CSO microcapsules using CS protein isolate and CS gum as the WM. The results showed that the SGF ranged from 38 to 79% and the SIF ranged from 15 to 23%. The anti-inflammatory impact of CSO and its blends were studied with a variety of vegetable oils to determine the anti-lipoxygenase activity with the in vitro methods. The results showed that CSO and its blends showed strong and differential anti–cancer activity^64^. Lavanya et al.^15^ also indicated that microcapsules of CO and FO were more stable with acidic conditions.

**Table 5:** Amount of oil released (%) from spray–dried microcapsules (SDM) with simulated gastric fluid (SGF) with and without heating.

Sample	Without heating	With heating
Chia and FO blended/un–encapsulated	64 ± 3^a^	70 ± 3^a^
SDM	42 ± 2^b^	63 ± 3^b^

*FO* Fish oil, *SDM* Spray–dried microcapsules.

Results are represented as mean ± standard deviation.

^a-b^Indicate different letters at the level of significance (*p* ≤ 0.05) in the same column.

**Table 6:** Chemical characterization of SDM samples after in vitro digestion.

Sample preparation	Saturated fatty acids	Monounsaturated fatty acids	Long chain PUFA
Crude chia and FO blend	49 ± 1^a^	18 ± 1^a^	42 ± 2^a^
SDM	52 ± 1^a^	19 ± 1^a^	43 ± 2^a^

*PUFA* Polyunsaturated fatty acids, *SDM* Spray–dried microcapsules, *FO* Fish oil, Results are represented as mean ± standard deviation.

^a^Indicate different letters at the level of significance (*p* ≤ 0.05) in the same column.

### Characteristics of fatty acids in SDM after in vitro digestion

In vitro digestion in the laboratory with appropriately controlled conditions^65^ suggested that the droplet adsorption layer and the droplet size have an important role in controlling the action of lipases and lipid digestion^66,67^. Similarly, several studies have focused on the influences of dietary lipid composition on gastrointestinal digestion. The majority of these studies have focused on the release rate of LCPUFA in the gastrointestinal tract^68^. In this study, in vitro digestion was evaluated by comparing the blend of CSO and FO with SDM. There were no significant changes in saturated fatty acids, monounsaturated fatty acids, and LCPUFA, whether or not they were exposed to gastrointestinal conditions. However, compared to the release of SDM, the value of saturated fatty acids increased slightly from 49 ± 1 to 52 ± 1% after digestion. In addition, a similar trend was observed for fatty acids and LCPUFA (Table 7). Therefore, it was concluded that the same amount of fatty acids was available after in vitro digestion whether encapsulated or not. Lavanya et al.^15^ indicated that microcapsules of CO and FO were more stable with acidic conditions. Goyal et al.^41^ showed a 20 to 23% oil release in the sequential digestion process with flaxseed oil.

**Table 7:** Amount of oil release in % of SDM with SGF + SIF (2 to 3 h).

Sample	RO (%)					
	Without heating			With heating		
	SGF incubation time (2 h)	SIF incubation time (3 h)	SGF + SIF incubation time (4 h)	SGF incubation time (2 h)	SIF incubation time (3 h)	SGF + SIF incubation time (4 h)
Crude chia and FO blend	14 ± 1^a^	14 ± 1^a^	15 ± 1^a^	20 ± 1^a^	21 ± 1^a^	22 ± 1^a^
SDM	12.2 ± 0.5^a^	12.3 ± 0.5^a^	12.4 ± 0.5^a^	17 ± 1^a^	19 ± 1^a^	21 ± 1^a^

*SDM* Spray–dried microcapsules, *SGF* Simulated gastric fluid, *SIF* Simulated intestinal fluid, *RO* Release of oil, *FO* Fish oil.

^a^Indicate different letters at the level of significance (*p* ≤ 0.05) in the same column.

## Conclusions

The main aim of this study was to retain the fatty acid profile and in vitro digestion of SDM. The maximum ALA, EPA, and DHA were observed with run 9. It was concluded that IAT was a major spray-drying factor that significantly impact the fatty acid profile of SDM compared to other spray-drying factors. FTIR and XRD demonstrated that the selected process and materials could successfully microencapsulate the chia/fish oil blend within the GA and MD. No significant changes were observed in saturated fatty acids, monounsaturated fatty acids, and LCPUFA, whether they were exposed or not to gastrointestinal conditions. Further studies should be focused on producing ω–3 enriched products for use as therapeutic agents for the treatment of multiple disorders. In vivo and cohort clinical trials are recommended.

## Data Availability

Data is contained within the article.
